# The impact of COVID-19 on smoking patterns in Pakistan: findings from a longitudinal survey of smokers

**DOI:** 10.1093/ntr/ntaa207

**Published:** 2020-10-08

**Authors:** Kamran Siddiqi, Faraz Siddiqui, Amina Khan, Saeed Ansaari, Mona Kanaan, Mariam Khokhar, Ziauddin Islam, Masuma Pervin Mishu, Linda Bauld

**Affiliations:** 1 Seebohm Rowntree Building, Department of Health Sciences, University of York, York, UK; 2 The Initiative, Orange Grove Farm, Main Korung Road, Banigala, Islamabad, Pakistan; 3 ARRC Building, Department of Health Sciences, University of York, York, UK; 4 Tobacco Control Cell, Ministry of National Health Services, Regulations and Coordination, Pakistan; 5 Usher Institute and SPECTRUM Consortium, College of Medicine and Veterinary Medicine, University of Edinburgh, EH8 9AG, Edinburgh, UK

## Abstract

**Introduction:**

We investigated the influence of COVID-19 on smoking patterns in Pakistan.

**Methods:**

In a longitudinal survey, we asked cigarette smokers in Pakistan about their smoking behaviours before and since COVID-19. Smokers were recruited before COVID-19 using two-stage random probability sampling. Since COVID-19, three subsequent waves were conducted over the telephone, asking additional questions on social determinants, mental health and wellbeing. Based on the first two waves, we estimated the proportion of smokers who stopped, decreased, maintained, or increased smoking. We also explored any factors associated with the change in smoking patterns. In those who stopped smoking soon after COVID-19, we estimated the proportion relapsed in subsequent waves. We estimated all proportions based on complete-case analysis.

**Results:**

We recruited 6,014 smokers between September 2019 and February 2020; of these, 2,087 (2,062 reported smoking outcomes) were followed up in May 2020 after COVID-19. Since COVID-19, 14% (290/2,062) smokers reported quitting. Among those who continued smoking: 68% (1210/1772) reduced, 14% (239/1772) maintained, and 18% (323/1772) increased cigarette consumption; 37% (351/938) reported at least one quit attempt; 41% (669/1619) were more motivated while 21% (333/1619) were less motivated to quit. Changes in smoking patterns varied with nicotine dependence, motivation to quit, and financial stability since COVID-19. Among those reporting quitting soon after COVID-19, 39% (81/206) relapsed in the subsequent months (June-July 2020).

**Conclusions:**

There have been significant bidirectional changes in smoking patterns since COVID-19 in Pakistan. While many people stopped, reduced, or tried quitting smoking, some increased smoking, and some relapsed after quitting.

**Implications:**

We observed significant and complex changes in people’s smoking patterns, which are likely to be attributable to the COVID-19 pandemic and replicated in similar events in the future. Assessing these changes are essential for most low- and middle-income countries like Pakistan, where the vast majority of tobacco users live, but cessation support is still rudimentary. If provided routinely, smoking cessation interventions can potentially support millions of highly motivated individuals in quitting successfully both in general as well as in global events like COVID-19, in particular.

## INTRODUCTION

Considered as the most significant challenge since World War II,[1] COVID-19 has infected over 24 million individuals worldwide so far. The pandemic and the subsequent measures e.g. social distancing, self-isolation, and lockdowns have led to business closures, loss of income,[2] and livelihoods, and a rise in mental distress.[3] With vast swathes of the global population worried about the virus, suffering from economic distress and living in social isolation, an impact on health behaviours is inevitable.[4] The trajectory of health risk behaviours such as alcohol consumption, smoking, physical activity, and dietary habits is important to document due to their long-term consequences. For example, even small scale changes in tobacco use consumed by over a billion people worldwide,[5] could lead to major shifts in future disease burden. More importantly, a better understanding of the magnitude and nature of such behaviour change can help services and policymakers to respond in an agile and effective manner.

The interaction between COVID-19 and smoking behaviour is likely to be complex and bidirectional. It is suggested that COVID-19 might have led to a reduction or stopping smoking in some but an increase in others.[6] The evidence on the effect of smoking on COVID-19 is still emerging;[7] with much uncertainty on whether smoking is linked with COVID-19 infection, hospitalisation or mortality.[8] In addition to such potential risks, access (business closures), affordability (loss of income) and opportunity (lockdowns) to smoke might have driven down its use.[9] Similarly, for some tobacco users, mental stress, anxiety, social isolation, and lack of access to cessation support might have resulted in increased consumption.[10] The evidence, mostly from high-income countries, suggests that while an unusual number of smokers have been motivated to quit during the pandemic[11] and have either cut down smoking or made quit attempts,[9] there are some who increased smoking.[12] There is little information so far on those quitting after the outbreak and maintaining abstinence. There is also little evidence on the impact of COVID-19 on smoking patterns in low- and middle-income countries (LMICs), where most of the world tobacco users now reside.[5] LMICs are at different stages of the tobacco epidemic, and some of the factors mediating between COVID-19 and smoking may act out differently in LMICs due to contextual differences.[13] In this study, we aimed to assess the impact of COVID-19 on smoking patterns in Pakistan; we also explored which factors might have influenced any change in smoking behaviour.

## METHODS

### Design

We conducted a longitudinal survey among regular cigarette smokers in the ten most populous cities of Pakistan. The first survey wave was conducted face-to-face, prior to the COVID-19 (Sept 2019-Feb 2020) outbreak in Pakistan (where the first case was recorded on March 12th) and the subsequent three waves soon after (May-July 2020). As lockdowns were imposed from 24th March 2020, ordering all public transport, markets, offices, shopping malls, restaurants, and public areas to be shut down, data for these subsequent waves were collected over the telephone.

### Participants and sampling methods

Before COVID-19 (wave 1), we recruited people aged 15+ who smoke at least 25 out of the last 30 days for at least six months. Households were identified using a two-stage random probability sampling. At the first stage, we used stratified random sampling to select 346 Union Councils (UCs) from the ten most populous cities. We assessed which households were eligible (at least one smoker aged 15+) in these UCs and then randomly selected 20 households at the second stage. One participant per household was identified using Kish Grid method[14] and recruited after informed consent.

### Data collection

Data were collected in password-protected digital tablets by field investigators in real-time. At wave 1, participants were asked about socio-demographic characteristics including age, education (no formal education, education till primary, secondary or high school, and graduation or above) and smoking behaviours including using Motivation To Stop Smoking (MTSS),[15] Strength of Urges to Smoke (SUTS)[16] and Heaviness of Smoking Index (HSI)[16] scales.

Since the COVID-19 outbreak in Pakistan, those who consented for follow-up over the phone were surveyed in three consecutive waves. To explore changes in their smoking behaviour during the pandemic, we asked the participants to choose from the following options: (i) I smoke cigarettes every day, (ii) I smoke cigarettes, but not every day, (iii) I smoke cigarettes, but I also smoke tobacco in other forms, (iv) I smoke tobacco, but I also use smokeless tobacco, (v) I have stopped smoking cigarettes, but I smoke other form(s) of tobacco (bidi/water pipe/cigar), (vi) I have stopped smoking tobacco, but I continue to use smokeless tobacco, (vii) I have stopped smoking tobacco but now I use smokeless tobacco, and (viii) I have stopped using all forms of tobacco. We also repeated MTSS and asked about the number of cigarettes/day and quit attempts made in the last six months. We used the above to define new variables and estimate proportions stopped, increased, maintained, or decreased cigarette smoking. Additional variables were created to identify those that stopped smoking cigarettes, this included individuals who continued other forms of tobacco smoking or smokeless tobacco.

We included additional derived variables at wave 2 e.g. housing security (concerned about eviction/non-payment of rent [Yes/No]), food security (had less food or went hungry [Yes/No]), status of the main earner (unemployed, employed but likely to lose job, employed and not likely to lose job), change in financial well-being in the past 3 months (worse off, about the same, better off), PHQ-9[17] for depression (none, mild, moderate, moderately severe, severe), GAD-7[18] for anxiety (none, mild, moderate, severe) and Warwick-Edinburgh Mental and Wellbeing Scale[19] for general health and well-being.

**Statistical Analysis**

A descriptive analysis reported changes in smoking behaviours during the pandemic, using data from participants who completed both the first and second waves. We estimated the overall proportion of smokers who quit smoking and those who decreased, maintained or increased their cigarette consumption since wave 1. We also estimated the proportion of quitters at wave 2 relapsing in subsequent waves. We estimated all proportions based on complete-case analysis. We explored associations between socio-demographic, smoking-related, psychosocial health and well-being measures and the change in smoking behaviours; we focussed on two sub-groups, i.e. those who reported complete abstinence and those reporting an increase in their cigarette consumption at wave 2. We reported bi-variate associations for age using independent student's t-test, and for categorical variables using chi-square test of independence. Alternatively, Fisher's exact test was used in comparisons where expected cell counts were <5. Statistical analyses were performed on STATA version 16.

Ethics approval for wave 1 was given on 20th June 2019 and for waves 2, 3 and 4 on 15th April 2020 by the National Bioethics Committee, Pakistan.

## RESULTS

We recruited 6,014 adult smokers between September 2019 and February 2020. Of these, contact details for a phone-based follow up were provided by 4,132 participants. We contacted 3,342 of these participants between 5th May 2020 and 4th June 2020 for wave 2. Data collection was carried out on 2,087 (62.5%) smokers - reasons for non-participation were incorrect telephone numbers (n=321), non-response (n=875), refusal to participate (n=46) and deaths (n=13). In the two subsequent waves, data were collected on 2,327 and 2,219 participants between 5th of June 2020 and 24th of June 2020 and between 25th of June 2020 and 15th of July 2020, respectively. The key socio-demographic and smoking characteristics of those followed up in comparison to those not followed-up are presented as *supplementary material*.

Of all participants (2,062 reported smoking outcomes) at wave 2, 14% (290/2062) stopped smoking cigarettes completely; 13.5% (279/2062) stopped using all forms of tobacco; 11 stopped smoking cigarettes but continued either smoking other forms of tobacco (6) or using smokeless tobacco (5). Of those who stopped smoking cigarettes at wave 2 and were followed-up in subsequent waves, 39.3% (81/206) relapsed either at wave 3 or 4. Among those who were still smoking at wave 2: 68% (1210/1772) reduced, 14% (239/1772) maintained, and 18% (323/1772) increased cigarette consumption at wave 2. Moreover, the mean number of cigarettes/day dropped from 14 to 9 cigs/day. Among those smoking at wave 2, 37% (351/938) made at least one quit attempt; 41% (669/1619) were more motivated and 21% (333/1619) were less motivated to quit according to MTSS.

Table 1 describes the distribution of socio-demographic and smoking variables (collected at wave 1) in those who were followed-up at wave 2; it also examines any statistically significant differences between those who stopped smoking vs. those who continued as well as between those who increased their smoking vs. the rest. Those who stopped smoking were more motivated to quit and were less nicotine dependent as per HSI and SUTS measured at wave 1 than those who continued. Those who increased their smoking were more likely to be single and on average younger and less dependent on tobacco than the rest of the cohort. These differences were statistically significant. Table 2 describes the distribution of social determinants, mental health and well-being variables (collected at wave 2) and assesses any statistically significant differences between the two categories i.e. those who stopped and those who increased smoking and the rest. More people who stopped smoking appear to be financially worse off and likely to lose their jobs as compared to those who continued smoking. These differences were statistically significant. On the contrary, those who increased smoking were a little more financially stable than those who didn't. On mental health

(GAD-7 and PHQ-9) and wellbeing (WEMWBS), we did not find any evidence of statistically significant differences between the groups.

## DISCUSSION

We report findings mainly from wave 2 of a longitudinal study in which smokers, recruited just before COVID-19, were followed up in successive waves to study changes in their smoking behaviour. We observed significant bidirectional changes. An estimated one in seven participants reported quitting after COVID-19 restrictions and among those who continued, more than two-thirds reduced their cigarette consumption. One-third made at least one quit attempt and 41% were more motivated to quit. Participants with lower nicotine dependency and greater financial concerns were more likely to quit than those who continued. On the other hand, 18% increased their smoking frequency, one in five smokers were less motivated to quit than before and 39% relapsed after quitting. Those who increased their smoking were less nicotine dependent and a little more financially stable than those who did not. No significant associations were found between changes in smoking behaviour and mental health and well-being.

Our findings are consistent with previous studies reporting a bidirectional effect of COVID-19 observed in smokers.[6,10,11] In one of these, Klemperer et al found that in response to COVID-19, the motivation to quit increased in 35.6% but reduced in 16.2% of smokers;[11] fear of COVID-19 was associated with a change in motivation. Likewise, 22.9% smokers made at least one quit attempt, 28.3% decreased their cigarette use, but another 30.3% increased its use since COVID-19.[11] In another group of cigar users, while two-third participants intended to quit and nearly half made a quit attempt, far more smokers reported increasing their smoking than those reporting a decline.[10]

Our paper describes self-reported changes in smoking behaviour in a large sample of a nationally representative cohort of smokers. To our knowledge, this is the only study that reports relapse rates among those who stopped soon after COVID-19 outbreak. However, there are several weaknesses that may limit its external validity. While the first wave of data collection finished just before COVID-19, it started a few months before the pandemic. It is possible that some of the reported changes might have happened before COVID-19. However, the scale of change is significant indicating an attribution to the outbreak. For example, the number of smokers making at least one quit attempt in the last six months reported at wave 2 (37%) was significantly higher than that reported for the last 12 months at wave 1 (28%) and Pakistan's Global Adult Tobacco Survey (25%). We didn't observe any structural changes that might have led to a disproportionate change in smoking patterns (e.g. tobacco taxes remained unchanged during this period). Seasonal variations can influence smoking behaviours but we were not able to confirm or adjust our estimates due to lack of local data. We were only able to reach a third of our cohort in the second wave. While this limits the external validity of our findings, the reasons for this non-response is unlikely to be linked to their smoking status except in those who refused (only 1.3% of everyone contacted). A comparison between those followed up in any of the three waves (2-4) and those not followed up revealed small but statistically significant differences in relation to sex, education status and addiction levels (see supplementary materials). Reasons for the changes in smoking behaviours were not recorded in this study; but relevant questions will be included in the future waves. We were only able to report short-term abstinence and the future waves can also assess long-term abstinence. Finally, self-reported smoking status is always less accurate than that validated biochemically, a limitation that we could not overcome in a telephone-based survey.

Smoking cessation treatment could have converted an unusually high number of quit attempts observed among smokers during COVID-19 into permanent abstinence. In a study in Wuhan, more people were successful in quitting (31.1%) following treatment than the success rates observed before COVID-19 (23.7%).[20] Most LMICs have very limited access to smoking cessation treatments. An increased interest in quitting in global events like COVID-19 further underlines the need to support those who are likely to quit. Healthcare professionals could use their patients' concern about COVID-19 as a 'teachable moment' to help them quit. Offering smoking cessation treatment by implementing Article 14 of the WHO Framework Convention for Tobacco Control should become an even greater priority for countries like Pakistan where millions of smokers are willing to quit but have little access to cessation interventions.

## ACKNOWLEDGEMENTS

We are grateful to our survey field team members for their dedication and hard work under challenging circumstances. Their names are as follows: Muhammad Imtiaz, Muhammad Shahzad, Khurram Abbas Khosa, M Shahid Khan, Musaddiq Hussain Ghauri, Muhammad Usman Jarral, Muhammad Danish Anjumn, Asad Taimoor, Mubasher Zia, Ifzal Nadeem, M Sohail Ijaz, M Asim Shazad, Sidra, Ali Raza,Qazi Amjad Ali, Wasim Akram, Zuhaib, M Asad Rafique, Rashid Maqbool, M Asim Zahoor, M Fahim Ansari, Khaista Gul, Sheraz Ali Yousaf Zai, Ghulam Sheryar, Mansoor Ahmed,Abdul rasheed, Adeel javaid, M Umer Shaheen, Hammad Akhtar, Ali Raza, Rameez Ali Khushk, Ali Akber Dolo, Tanveer Ahmed, Jashvant, Bilal ahmed, Sir Syed Ahmed, Karima Bibi, Khursheed Bibi, Nazeera Rahman, Fahmeeda, Shamsa, Atiqa, Rabel, Tariq Ahmed, Uroosa, Abid Taj, Amar Kumar, Faizan Ahmed, Mohd Rizvi, Hina Sharif, Javed Ahmed, Saleem Raza, Mehreen, Sanam Abbas, Shazia, Taranum

## Funding

Wave 2 of the survey was funded by the University of Edinburgh's Scottish Funding Council Global Challenges Research Fund (GCRF) allocation and made possible through the existing GCRF project the Tobacco Control Capacity Programme (Grant Ref: MR/P027946/1). Four authors (KS, FS, MK, MPM) were partially funded by NIHR Global Health Group ASTRA - Addressing Smokeless Tobacco and building Research capacity in south Asia (Grant ref: 17/63/76).

## References

1. Global Cooperation Must Adapt to Meet Biggest Threat since Second World War, Secretary-General Says on International Day, as COVID-19 Transcends Borders. Accessed August 3, 2020. https://www.un.org/press/en/2020/sgsm20058.doc.htm

2. Impact of COVID-19 on financial lives in 8 countries over 2 weeks. Published April 23, 2020. Accessed August 3, 2020. https://bfaglobal.com/covid-19/insights/the-impact-of-covid-19-on-financial-lives/

3. Pierce M, Hope H, Ford T, et al. Mental health before and during the COVID-19 pandemic: a longitudinal probability sample survey of the UK population. *Lancet Psychiatry*. Published online July 21, 2020. doi:10.1016/S2215-0366(20)30308-4

4. Arora T, Grey I. Health behaviour changes during COVID-19 and the potential consequences: A mini-review. *J Health Psychol*. 2020;25(9):1155-1163.

5. *Smoking Prevalence and Attributable Disease Burden in 195 Countries and Territories, 1990–2015: A Systematic Analysis from the Global Burden of Disease Study 2015*. Elsevier; 2017.

6. Bommele J, Hopman P, Walters BH, et al. The double-edged relationship between COVID-19 stress and smoking: Implications for smoking cessation. *Tob Induc Dis*. 2020;18:63.

7. Edwards R, Munafò M. COVID-19 and Tobacco: More Questions Than Answers. *Nicotine & Tobacco Research*. Published online 2020. doi:10.1093/ntr/ntaa132

8. Simons D, Shahab L, Brown J, Perski O. The association of smoking status with SARS-CoV-2 infection, hospitalisation and mortality from COVID-19: A living rapid evidence review. *Qeios*. Published online 2020. https://www.qeios.com/read/UJR2AW.3

9. Tattan-Birch H, Perski O, Jackson SE, Shahab L, West R, Brown J. COVID-19, smoking, vaping and quitting: A representative population survey in England. *medRxiv*. Published online 2020. https://www.medrxiv.org/content/10.1101/2020.06.29.20142661v1.abstract

10. Kowitt SD, Cornacchione Ross J, Jarman KL, et al. Tobacco Quit Intentions and Behaviors among Cigar Smokers in the United States in Response to COVID-19. *Int J Environ Res Public Health*. 2020;17(15). doi:10.3390/ijerph17155368

11. Klemperer EM, West JC, Peasley-Miklus C, Villanti AC. Change in tobacco and electronic cigarette use and motivation to quit in response to COVID-19. *Nicotine Tob Res*. Published online April 28, 2020. doi:10.1093/ntr/ntaa072

12. Stanton R, To QG, Khalesi S, et al. Depression, Anxiety and Stress during COVID-19: Associations with Changes in Physical Activity, Sleep, Tobacco and Alcohol Use in Australian Adults. *Int J Environ Res Public Health*. 2020;17(11). doi:10.3390/ijerph17114065

13. Hopman J, Allegranzi B, Mehtar S. Managing COVID-19 in Low- and Middle-Income Countries. *JAMA*. Published online March 16, 2020. doi:10.1001/jama.2020.4169

14. McBurney P. On Transferring Statistical Techniques Across Cultures: The Kish Grid. *Curr Anthropol*. 1988;29(2):323-325.

15. Kotz D, Brown J, West R. Predictive validity of the Motivation To Stop Scale (MTSS): a single-item measure of motivation to stop smoking. *Drug Alcohol Depend*. 2013;128(1-2):15-19.

16. Fidler JA, Shahab L, West R. Strength of urges to smoke as a measure of severity of cigarette dependence: comparison with the Fagerström Test for Nicotine Dependence and its components. *Addiction*. 2011;106(3):631-638.

17. Kroenke K, Spitzer RL, Williams JBW. The PHQ-9: validity of a brief depression severity measure. *J Gen Intern Med*. 2001;16(9):606-613.

18. Spitzer RL, Kroenke K, Williams JBW, Löwe B. A brief measure for assessing generalized anxiety disorder: the GAD-7. *Arch Intern Med*. 2006;166(10):1092-1097.

19. Tennant R, Hiller L, Fishwick R, et al. The Warwick-Edinburgh Mental Well-being Scale (WEMWBS): development and UK validation. *Health Qual Life Outcomes*. 2007;5:63.

20. Kayhan Tetik B, Gedik Tekinemre I, Taş S. The Effect of the COVID-19 Pandemic on Smoking Cessation Success. *J Community Health*. Published online July 8, 2020. doi:10.1007/s10900-020-00880-2

Table 1: Socio-demographic and smoking characteristics of participants and their smoking behaviour at wave 2 of the survey (n=2062). Counts and (%) are presented unless specified

| | **Total sample** | **Quit smoking** | **Continued smoking** | **P value** | **Increased smoking** | **Didn't increase smoking** | **p value** |
|---|---|---|---|---|---|---|---|
| | 2062 | 290 | 1772 | | 323 | 1739 | |
| **Age** (in years)* (mean±SD) | 45.0 ±14.5 | 45.7±16.1 | 44.9.1±14.2 | 0.39 | 43.4±15.3 | 45.3±14.3 | 0.03 |
| **Education** | | | | | | | |
| No formal education | 567 (27.5) | 99 (34.2) | 468 (25.6) | 0.06** | 88 (27.2) | 479 (27.5) | 0.19 ** |
| Up to primary education | 485 (23.5) | 57 (19.6) | 428 (22.3) | | 93 (28.8) | 392 (22.5) | |
| Up to secondary education | 588 (28.5) | 78 (26.9) | 510 (28.0) | | 99 (30.6) | 489 (28.1) | |
| High school | 177 (8.6) | 23 (7.9) | 154 (9.6) | | 24 (7.4) | 153 (8.9) | |
| Graduation or above | 148 (7.2) | 24 (8.3) | 124 (7.4) | | 18 (5.6) | 130 (7.5) | |
| *Missing* | 97 (4.7) | 9 (3.1) | 88 (7.1) | | 1 (0.4) | 96 (5.5) | |
| **Marital status** | | | | | | | |
| Single | 267 (12.9) | 41 (14.1) | 226 (12.75) | 0.34** | 62 (19.2) | 205 (11.8) | 0.003** |

| | | | | | | | |
|---|---|---|---|---|---|---|---|
| Married | 1644 (79.7) | 231 (79.7) | 1413 (79.7) | | 249 (77.1) | 1395 (80.2) | |
| Separated/divorced/widowed | 57 (2.7) | 10 (3.4) | 47 (2.6) | | 12 (3.7) | 45 (2.6) | |
| *missing* | 94 (4.5) | 8 (2.8) | 86 (4.85) | | 0 (0) | 94 (5.4) | |
| **Heaviness of Smoking Index** | | | | | | | |
| Low | 337 (16.3) | 43 (14.8) | 294 (16.6) | 0.009** | 44 (13.6) | 293 (16.8) | <0.001** |
| Medium | 1291 (62.6) | 190 (65.5) | 1101 (62.1) | | 227 (70.3) | 1064 (61.2) | |
| High | 203 (9.8) | 14 (4.8) | 189 (10.7) | | 7 (2.2) | 196 (11.3) | |
| Missing | 231 (11.2) | 43 (14.8) | 188 (10.6) | | 45 (13.9) | 186 (10.7) | |
| (mean±SD) | 2.5±1.0 | 2.4±0.8 | 2.5±1.0 | | 2.4±0.9 | 2.4±1.0 | |
| **Strength of Urge To Smoke** | | | | | | | |
| None | 140 (6.8) | 37 (12.7) | 103 (5.8) | <0.001** | 44 (13.6) | 96 (5.5) | <0.001** |
| Slight | 339 (16.4) | 78 (26.9) | 261 (14.7) | | 100 (30.9) | 239 (13.7) | |
| Moderate | 797(38.6) | 94 (32.4) | 703 (39.6) | | 121 (37.5) | 676 (38.9) | |
| Strong | 479 (23.2) | 53 (18.3) | 426 (24.0) | | 41 (12.7) | 438 (25.2) | |
| Very strong | 133 (6.4) | 15 (5.2) | 118 (6.6) | | 13 (4.0) | 120 (6.9) | |
| Extremely strong | 80 (3.8) | 5 (1.7) | 75 (4.2) | | 4 (1.2) | 76 (4.4) | |
| missing | 94(4.5) | 8 (2.7) | 86 (4.8) | | 0 (0) | 94 (5.4) | |
| (mean±SD) | 2.2 ±1.1 | 1.8±1.1 | 2.2±1.1 | | 1.66±1.0 | 2.3±1.1 | |

| | | | | | | | |
|---|---|---|---|---|---|---|---|
| **Motivation to stop smoking** | | | | | | | |
| No desire or intention to quit | 904 (43.8) | 119 (41.0) | 785 (44.3) | 0.009** | 141 (43.7) | 763 (43.9) | 0.65** |
| Desire to quit | 764 (37.0) | 100 (34.5) | 664 (37.4) | | 132 (40.9) | 632 (36.3) | |
| Desire and intention to quit | 279 (13.5) | 56 (19.3) | 223 (12.5) | | 46 (14.2) | 233 (13.4) | |
| missing | 115 (5.6) | 15 (5.2) | 100 (5.6) | | 4 (1.2) | 111 (6.4) | |

* Student's t-test

** test statistics represent only non-missing values

Table 2. Social determinants, mental health and well-being of participants and their smoking behaviour at wave 2 of the survey (n=2062). Counts and (%) are presented unless specified.

| | Overall | Quit smoking | Continued smoking | p value | Increased smoking | Didn't increase smoking | p value |
|---|---|---|---|---|---|---|---|
| **Food security** | | | | | | | |
| No | 661 (32.1) | 92 (31.7) | 1203 (67.9) | 0.89 | 84 (26.0) | 577 (33.1) | 0.01 |
| Yes | 1401 (67.9) | 198 (68.3) | 569 (32.1) | | 239 (74.0) | 1162(66.9) | |
| **Housing security** | | | | | | | |
| No | 681 (33.1) | 73 (25.2) | 1164 (65.7) | 0.002 | 112 (34.7) | 569 (32.7) | 0.49 |
| Yes | 1381 (66.9) | 217 (74.8) | 608 (34.3) | | 211 (65.3) | 1170 (67.3) | |
| **Financial situation since 3 months ago** | | | | | | | |
| Worse | 1454 (70.5) | 221 (76.2) | 1233 (69.6) | <0.001 | 205 (63.5) | 1249 (71.8) | 0.01 |
| Better/same as before | 484 (23.5) | 67 (23.1) | 417 (23.5) | | 94 (29.1) | 390 (22.4) | |
| Don't know/don't wish to answer | 124 (6.0) | 2 (0.7) | 122 (6.9) | | 24 (7.4) | 100 (5.8) | |
| **The main earner** | | | | | | | |
| Unemployed | 703 (34.1) | 100 (34.5) | 603 (34.0) | 0.009** | 104 (32.2) | 599 (34.4) | 0.01** |
| Employed, likely to lose job | 352 (17.0) | 65 (22.4) | 287 (16.2) | | 39 (12.1) | 313 (18.0) | |
| Employed, unlikely to lose job | 956 (46.4) | 114 (39.3) | 842 (47.5) | | 172 (53.2) | 784 (45.1) | |

| | | | | | | | |
|---|---|---|---|---|---|---|---|
| Missing | 51 (2.5) | 11 (3.8) | 40 (2.2) | | 8 (2.5) | 43 (2.5) | |
| **Health status** | | | | | | | |
| Good | 1798 (87.2) | 254 (87.6) | 1544 (87.1) | 0.98 | 277 (85.8) | 1521(87.5) | 0.04 |
| Fair | 220 (10.7) | 31 (10.7) | 189 (10.7) | | 40 (12.4) | 180 (10.4) | |
| Poor | 38 (1.8) | 5 (1.7) | 33 (1.8) | | 3 (0.9) | 35 (2.0) | |
| Don't know/Don't wish to answer | 6 (0.3) | 0 (0) | 6 (0.3) | | 3 (0.9) | 3 (0.1) | |
| **Anxiety (GAD 7)** | | | | | | | |
| None | 1001 (48.6) | 149 (51.6) | 846 (47.9) | 0.39 | 150 (46.4) | 851 (48.9) | 0.72 |
| Mild | 763 (37.0) | 94 (32.5) | 669 (37.9) | | 128 (39.6) | 635 (36.5) | |
| Moderate | 200 (9.7) | 31 (10.7) | 169 (9.6) | | 29 (8.9) | 171 (9.8) | |
| Severe | 97 (4.7) | 15 (5.2) | 82 (4.6) | | 16 (4.9) | 81 (4.6) | |
| Mean ±SD | 5.0±4.3 | 4.8±4.5 | 5.0±4.3 | | 5.0±4.4 | 4.9±4.3 | |
| **Depression (PHQ-9)** | | | | | | | |
| Minimal/non depressed | 1238 (60.1) | 178 (62.0) | 1054 (59.7) | 0.14 | 202 (62.5) | 1036 (59.7) | 0.15 |
| Mild | 518 (25.2) | 72 (25.1) | 446 (25.2) | | 69 (21.4) | 449 (25.9) | |
| Moderate | 213 (10.3) | 20 (6.9) | 193 (10.9) | | 42 (13.0) | 171 (9.8) | |
| Moderately severe | 71 (3.5) | 12 (4.2) | 59 (3.3) | | 8 (2.5) | 63 (3.6) | |

| | | | | | | | |
|---|---|---|---|---|---|---|---|
| Severe<br>Mean ±SD | 19 (0.9)<br>4.4±4.8 | 5 (1.7)<br>4.5±5.34.8 | 14 (0.8)<br>4.4±4.8 | | 2 (0.6)<br>4.3±4.8 | 17 (1.0)<br>4.5±4.8 | |
| **Warwick-Edinburgh mental and well being scores (WEMWBS)** Mean ±SD | 22.7±4.6 | 23.2±4.7 | 22.5±4.6 | 0.05* | 22.7±4.5 | 22.7±4.6 | 0.94* |

* Student's t-test

** test statistics represent only non-missing values
